# *Food-pics*: an image database for experimental research on eating and appetite

**DOI:** 10.3389/fpsyg.2014.00617

**Published:** 2014-06-24

**Authors:** Jens Blechert, Adrian Meule, Niko A. Busch, Kathrin Ohla

**Affiliations:** ^1^Division of Clinical Psychology, Psychotherapy and Health Psychology, University of SalzburgSalzburg, Austria; ^2^Institute of Psychology, University of WürzburgWürzburg, Germany; ^3^Hospital for Child and Adolescent Psychiatry, LWL University Hospital of the Ruhr University BochumHamm, Germany; ^4^Institute of Medical Psychology, Charité–UniversitätsmedizinBerlin, Germany; ^5^Berlin School of Mind and Brain, Humboldt Universität zu BerlinBerlin, Germany; ^6^Section Psychophysiology, Department of Molecular Genetics, German Institute of Human Nutrition Potsdam-RehbrückeNuthetal, Germany

**Keywords:** standardized food images, food pictures, food-cues, image properties, ERP, fMRI, eating behavior, obesity

## Abstract

Our current environment is characterized by the omnipresence of food cues. The sight and smell of real foods, but also graphically depictions of appetizing foods, can guide our eating behavior, for example, by eliciting food craving and influencing food choice. The relevance of visual food cues on human information processing has been demonstrated by a growing body of studies employing food images across the disciplines of psychology, medicine, and neuroscience. However, currently used food image sets vary considerably across laboratories and image characteristics (contrast, brightness, etc.) and food composition (calories, macronutrients, etc.) are often unspecified. These factors might have contributed to some of the inconsistencies of this research. To remedy this, we developed *food-pics*, a picture database comprising 568 food images and 315 non-food images along with detailed meta-data. A total of *N* = 1988 individuals with large variance in age and weight from German speaking countries and North America provided normative ratings of valence, arousal, palatability, desire to eat, recognizability and visual complexity. Furthermore, data on macronutrients (g), energy density (kcal), and physical image characteristics (color composition, contrast, brightness, size, complexity) are provided. The *food-pics* image database is freely available under the creative commons license with the hope that the set will facilitate standardization and comparability across studies and advance experimental research on the determinants of eating behavior.

## Introduction

Our current environment is characterized by frequent cues for highly palatable foods. Many researchers partially attribute rising obesity rates and problems in eating-related self-regulation to this factor (Meule and Vögele, 2013). To examine the factors underlying appetitive responses to foods, research is increasingly using food images (Van Der Laan et al., 2011). Visual food cues constitute, like odors, a primary sensory input that allows predictions about the edibility and palatability of a food object. Thus, visual food cues can be regarded conditioned stimuli that are associated with the hedonic and homeostatic effects of ingestion and are therefore themselves rewarding (Dagher, 2012). Also, overt eating behaviors are under strong conscious control and therefore do not always reveal underlying response tendencies. Using food images, neurocognitive and indirect measures have been particularly successful in the study of subtle appetitive and regulatory determinants of overt eating behavior.

The “picture viewing approach” is validated by several lines of evidence. *First*, food deprivation/hunger affects the response to food images on several levels. Short term food deprivation affects responses to food pictures as demonstrated for implicit food evaluation (e.g., Seibt et al., 2007; Hoefling and Strack, 2008), salivation (e.g., Wooley and Wooley, 1981), autonomic responding (e.g., Rodriguez et al., 2005), visual attentional processing (e.g., Stockburger et al., 2009b) and neural reward system activity (e.g., Labar et al., 2001; Uher et al., 2006; Castellanos et al., 2009; Goldstone et al., 2009). *Second*, food image responses reliably differentiate individuals with abnormal eating behavior from healthy controls: altered food cue processing has been reported in individuals with restrained (Blechert et al., 2010; Burger and Stice, 2011), external (Nijs et al., 2009) or emotional eating (Bohon et al., 2009), as well as in patients with eating disorders (Blechert et al., 2011; Nikendei et al., 2012) or obesity (Nijs and Franken, 2012; Martens et al., 2013). *Third*, food picture viewing tasks have been adapted to train eating control, for example, through food-specific inhibition tasks (e.g., stop-signal task; Van Koningsbruggen et al., 2013) or attentional retraining (Werthmann et al., 2013; Kakoschke et al., 2014; Kemps et al., 2014) with measurable effects on actual food intake, supporting the notion that responding to food images is causally involved in eating behavior. In sum, there is good evidence that the food picture viewing approach is a useful tool for the study of eating behavior and appetitive/motivational brain systems. However, a number of factors need to be taken into account during selection of images to be able to draw firm conclusions. These factors broadly fall into the categories (1) *food types*, (2) *individual differences*, and (3) *image characteristics*.

Regarding *food types*, cultures around the world have brought about a vast variety of foods that researchers need to consider when designing experiments. What might be the dimensions that need to be considered during image selection? First, foods differ in *caloric content*, which has been shown to affect early electrocortical responses (Toepel et al., 2009; Meule et al., 2013). However, caloric density often goes along with the degree of *food processing*: processed foods are often more energy-dense than natural, unprocessed foods. Processed foods furthermore differ in their *colors* from whole foods like fruits and vegetables, which utilize the entire color spectrum. Thus, image selection according to caloric density should simultaneously consider level of processing and colors. Besides caloric density, *macronutrients*, that are proteins, fats, and carbohydrates, should be taken into account, if craving for certain types of food is a construct of interest (e.g., craving for carbohydrates, Corsica and Spring, 2008). Furthermore, there are distinguishable food *classes* such as vegetables, meat-containing dishes, fruits, and snacks which each differ in their (seasonal) availability, readiness to eat, flavor, nutritional composition, healthiness, color, and familiarity. Obviously, the categorization of foods into some classes is dependent not only on individual experiences and availability of certain foods but also on the research questions asked. It is for that reason, that *food-pics* provides a variety of food images that cover many food classes and that, most importantly, can be classified as needed by the user.

Not only is there a wide variety of food types to choose from but researchers need to consider the targeted population and therefore *individual differences* for image selection. For example, if vegetarians or vegans are part of the sample, meat containing images should probably be avoided as these trigger altered neural and behavioral responses in vegetarians compared to omnivores (Stockburger et al., 2009a). Similar considerations apply to food preferences based on cultural, religious or health grounds (Hoffman et al., 2013). Individual preferences affect brain responses, which is why some studies individualize stimuli to match each participant's preferences (e.g., Hollmann et al., 2012; Giuliani et al., 2013). Further individual differences in age and gender, educational status, and body mass index (BMI) should be considered for images selection (Caine-Bish and Scheule, 2009; Raffensperger et al., 2010; Berthoud and Zheng, 2012).

A third class of factors are *image characteristics*. Unfortunately, dimensions such as brightness, contrast, or spatial frequencies have not received much attention in studies using food-related images. However, effects of such image features on visual perception and stimulus-evoked neuronal responses are well known. Consequently, it is recommended to carefully control the physical properties of visual stimulus material (Knebel et al., 2008; Willenbockel et al., 2010; Kovalenko et al., 2012; Ball et al., 2013). For example, the role of image complexity and spatial frequencies for neural responses are heavily debated in the field of face processing (Vuilleumier et al., 2003; Thierry et al., 2007; Rossion and Jacques, 2008) and are increasingly considered during affective picture viewing (Bradley et al., 2007; Delplanque et al., 2007; Wiens et al., 2011). Thus, similar standards must apply to studies using food pictures rendering standardized stimulus sets and associated meta-data essential. To this end, Foroni et al. (2013) recently presented an image database featuring food (natural, transformed), rotten food, non-food objects (natural, artificial), animals, and scenes along with normative ratings by 73 healthy participants and physical image properties (size, brightness and spatial frequency). They focused on the natural (food, non-food) and artificial distinction in their data analyses. While their database (Foodcast Research Image Database, FRIDa) represents an important step forward in the field of food picture research and their variety of images is broad, the number of edible food items is relatively restricted and, in part, specific to the Mediterranean cuisine. Moreover, their normative data stem from a small sample with little demographic diversity, resulting in a relatively low number of ratings per image.

In the present study, we present *food-pics*, a stimulus set of 568 food and 315 non-food images. In our study design (normative ratings, image characteristics), we aimed to be complementary to FRIDa and at the same time address some of its limitations. *Food-pics* was aggregated to represent a wider range of foods to allow applicability in western countries. Our normative participant samples (*N* = 1988) were selected to represent typical university student samples but, in total, span a comparatively wide range of age (11–77 years), BMI (12–67 kg/m^2^), and cultural background (German-speaking countries and the USA), to provide robust and generalizable normative data on commonly used perceptual and psychological parameters like palatability, desire to eat, recognizability, familiarity, valence and arousal. Physical image characteristics, that is, color, size, contrast, brightness, and complexity, were computed to complement the dataset and allow the selection of physically matching groups of images. Our analyses explore several example dimensions relevant to study design: (1) *image type* (e.g., food vs. non-food images) and *food type* (e.g., vegetables vs. meat vs. fruits, high- vs. low-calorie dense food, sweet vs. savory food, whole vs. processed food), (2) *individual differences* (e.g., demographics such as age, gender, and BMI, but also cultural background and vegetarianism) and (3) *state variables* (e.g., hunger and current weight reduction diet) on image ratings. We also explored the relationship of (4) *image characteristics* (e.g., contrast, brightness, complexity) with subjective ratings and nutritional content.

## Methods

### Stimuli

The database comprises 568 food images including sweet (e.g., ice cream, chocolate), savory (e.g., pistachios, sandwiches), processed (e.g., hamburger, French fries, potato chips, chocolate bars) and whole foods (e.g., vegetables and fruits) and beverages (e.g., coffee, orange juice). Images of single items (e.g., one apple), several items (e.g., three apples) as well as full meals (e.g., roast beef with vegetables), were included. The food images are complemented by 315 non-food images comprising animals (*n* = 37, e.g., butterflies, dogs), flowers and leaves (*n* = 42), common household objects (*n* = 89, e.g., bucket, flat iron), office supply (*n* = 20, e.g., paper clip, ball pen), kitchen accessories (*n* = 46, e.g., toaster, pan), as well as tools (*n* = 23, e.g., pliers, screws), food packaging (*n* = 33, e.g., pizza box; no food visible on packaging), and other objects (*n* = 25). Images were selected from a commercially available database (Hemera Photo Objects, Vols. I-III), collected from non-copyrighted sources on the internet, or taken in our lab using an Olympus SZ-31MR digital camera (OlympusCorp., Tokyo, Japan). All images are color photographs with a resolution of 600 × 450 pixels (96 dpi, sRGB color format). Images were standardized on background color (white) and selected/edited to be relatively homogeneous with regard to, viewing distance (≈80 cm), angle and simple figure-ground composition. The background was adapted to meet eating conditions: some foods can be presented without dishware (e.g., fruits or hamburger), while others naturally require a plate or bowl (e.g., soup or fruit salad).

### Image characteristics

**Figure 1:**
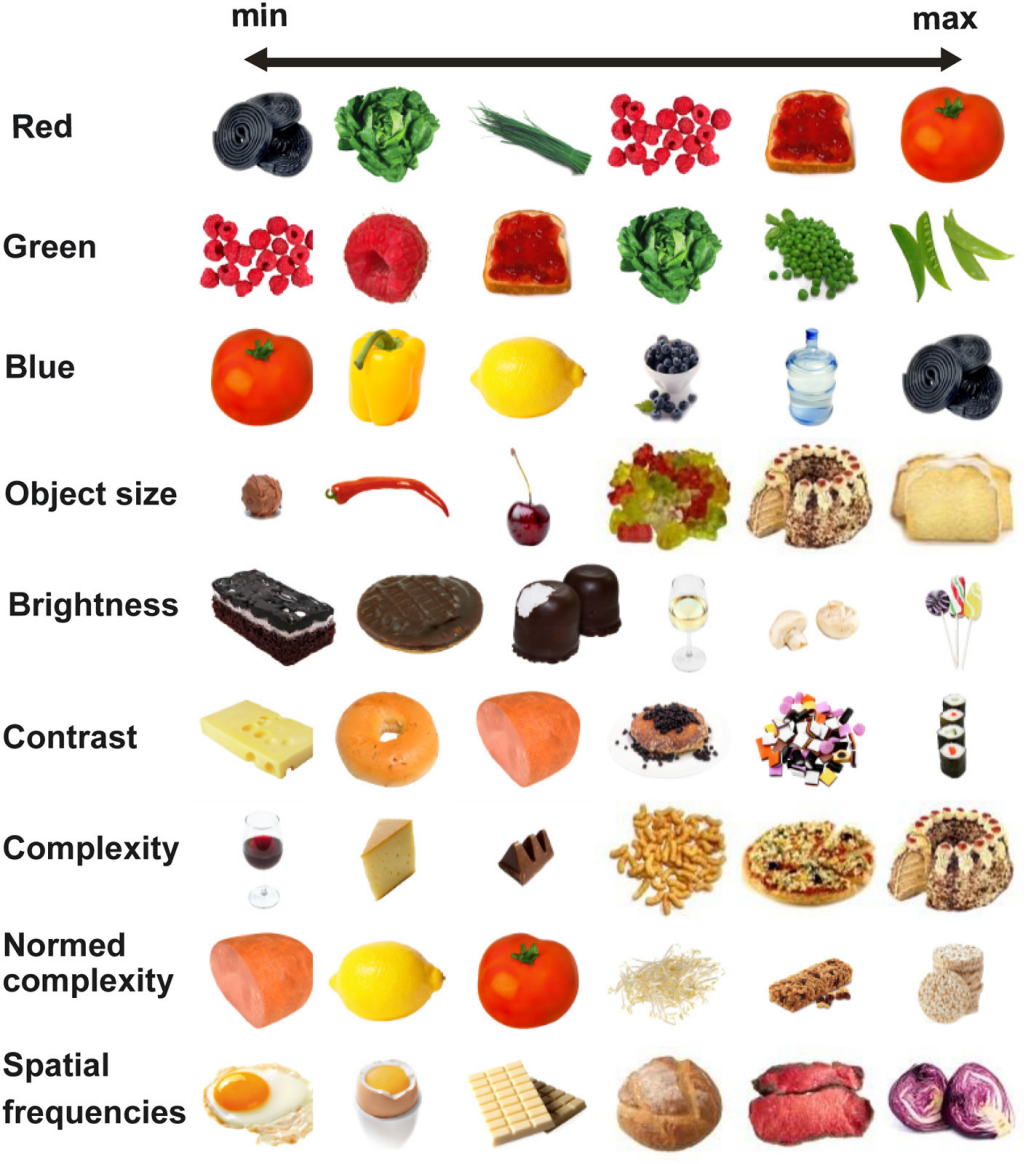
**Example pictures illustrating image characteristics from low (left) to high parameter value (right)**.

For each image, we computed relevant image properties that characterize the images' physical appearance using customized scripts written in Matlab R2011b (The Mathworks, Inc. Natick, USA). Scripts can be downloaded from the *food-pics* website (www.food-pics.sbg.ac.at). With the exception of the RGB channel contribution, all properties were computed after converting the colored image to gray values by forming a weighted sum of the red, green, and blue color channels: 0.2989 × red + 0.5870 × green + 0.1140 × blue. This procedure converts RGB images to gray-scale by eliminating the hue and saturation information while retaining image luminance (Poynton, 2012). The following image properties were analyzed:

*Color*, quantified as the proportional contribution of the red, green, and blue channel, averaged across all non-white pixels. For example, a tomato is characterized by a strong contribution of the red channel (see Figure 1).

*Size*, quantified as the proportion of non-white pixels relative to total number of pixels (identical as in Foroni et al., 2013).

*Brightness*, quantified as the difference between the mean luminance of all non-white pixels of the gray scale image and the white background (Foroni et al., 2013). Thus, the most salient objects (i.e., very dark objects on white background) yielded the highest brightness values.

*Within-object contrast*, quantified as the standard deviation of luminance across all non-white pixels of the gray scaled image. For example, an image of a black chocolate bar on a white plate contains pixels with luminance values ranging from very dark to white. Thus, this image is characterized by a high standard deviation of luminance values. By contrast, an image of whipped cream on a white plate comprises very few dark pixels, and so is characterized by a small standard deviation.

#### Spatial frequencies

Median power of each object was analyzed by computing a two-dimensional fast Fourier transform on the gray-scale images. One-dimensional power spectra were obtained by computing a radial average of the two-dimensional power spectra. This procedure yields a measure of the image's spatial frequencies, reflecting variations in pixel luminance, independent of their location in the image. To represent spectral power in a single value for each image, we computed the median power across all spatial frequencies.

#### Complexity

While some images display a single homogenous object (e.g., a slice of cheese), other images display multiple objects (e.g., an assortment of different fruits) or objects consisting of multiple components (e.g., a pizza). Images that are complex in this sense are characterized by multiple object outlines. Thus, we analyzed the images for outlines using a Canny edge detection algorithm (Canny, 1986) and quantified image complexity by computing the proportion of outline-related pixels within the image. However, the number of outline-pixels is also determined by the object's size—a magnified version of the identical object would have larger outlines and would yield a higher complexity value. Therefore, we also computed a *normalized complexity* measure that is independent of object size, by additionally dividing the proportion of outline-related pixels by the total number of non-white pixels in the image. Size and brightness were computed in the same way as reported by Foroni et al. (2013).

### Macronutrients

Number of kcal and macronutrient composition (proteins, carbohydrates, fat) of a depicted food were estimated for each food image by a trained research assistant (psychology master level student) using food databases on the internet and food packaging information. Kcal and macronutrients are provided as kcal/100g and grams/100g as well as total kcal and grams, respectively, for the depicted portion. Whenever multiple food items were displayed (e.g., grapes) counts were provided to facilitate analyses of experimental test meals. To cross-validate the accuracy of these data, a second research assistant (also a psychology master level student) estimated these data a second time for a randomly selected subsample of 38 food items^1^. Agreement between the two coders was excellent; Pearson correlations ranged from *r* = 0.84 to *r* = 0.99 with a mean of *r* = 0.95.

### Participants

Four samples completed an anonymous online survey (see Table 1 for sample descriptions) to provide normative data for *food-pics*. Only participants who completed all ratings for at least 3 food images were included (see “Online Survey” below). The first sample (“*UniHagen sample*,” *n* = 638) comprised undergraduates of the University of Hagen, a German distance teaching university, who completed the survey in exchange for course credit and the option of participating in a raffle for 3 × 50 Euro upon completion. The second sample was recruited through mailing lists of several universities in Germany, Switzerland and Austria (“*German-speaking sample*,” *n* = 831). The third sample addressed US-participants (“*US sample*,” *n* = 496), recruited though the online work marketplace “Mechanical Turk” at Amazon, where registered users work on online tasks in exchange for payment. The fourth sample addressed children and youth at an Austrian high school (“*Children/youth sample*,” *n* = 23) to extend the age range. The German-speaking and the Children/youth samples were also offered participation in a raffle for 3 × 50 Euros. All surveys were completed between May and August 2013. The ethics board of the University of Salzburg had approved the study.

**Table 1:** **Demographic characteristics by sample**.

	**German-speaking sample**	**US-American Sample**	**UniHagen**	**Austrian children and youth**
**AGE**				
Mean (*SD*)	24.7 (5.46)	35.9 (13.41)	32.8 (10.07)	13.9 (1.56)
Median (Min, Max)	23 (18–65)	32 (18–77)	30 (17–73)	14 (11–18)
**GENDER**				
Male (%)	16.7	36.3	17.2	60.9
**NATIONALITY (%)**				
Germany	93.0	0	93.1	4.35
Austria	3.01	0	2.35	91.3
Switzerland	0.12	0	1.1	0
Other European country	2.17	0	2.19	4.35
Non-European country	1.68	0	1.25	0
USA	0	98.7	0	0
Canadian	0	0.4	0	0
Other	0	0.80	0	0
**BODY MASS INDEX (kg/m^2^)**				
Mean(*SD*)	22.5 (3.70)	27.3 (7.29)	23.4 (4.68)	18.7 (2.77)
Median (Min, Max)	21.7 (14.2–45.3)	25.7 (15.5–67.4)	22.4 (12.1–60.5)	18.6 (14.6–24.34)
**EATING STYLE (%)**				
Omnivore	75.9	92.3	77.7	95.7
Vegetarian	20.2	5.4	19.6	4.3
Vegan	3.9	2.2	2.7	0.0
**CURRENTLY DIETING**				
(%)	10.3	23.8	9.9	4.3
**EMPLOYMENT (%)**				
High school	1.2	10.7	0.0	100
College/University	86.8	11.3	100	0.0
Apprenticeship	5.1	0.6	0.0	0.0
Self-employed	1.1	21.6	0.0	0.0
Unemployed	1.2	17.1	0.0	0.0
Other	4.6	38.7	0.0	0.0
**PROGRESS IN SURVEY**				
% Completed	77.6	69.6	89.8	78.3
% Partial completion	22.4	30.4	10.2	21.7

### Online survey

As participants could not be expected to reliably rate all 882 images, each participant rated a random subset of images, separately drawn from non-food and food images. Due to different modes of compensation (course credit, payment, raffle) the samples differed in the number of images rated by each participant: UniHagen sample 40 non-foods/80 foods, German-speaking sample 25/40, US sample 17/35, and Children/Youth sample 5/35. On average, each image was rated by 48.8 (*SD* = 22.9) participants.

The survey commenced with an assessment of demographics (age, gender, height, occupation, nationality) and eating habits (weight, diet: omnivore/vegetarian/vegan, weight-loss dieting) before displaying a detailed explanation as well as an example rating for all scales. During the survey, one image was displayed at a time and ratings were required for the dichotomous item *familiarity* (yes or no) and *recognizability* (easy or difficult). Visual analog scales (VAS, approximately 8 cm long) were displayed to rate *complexity* (only the extremes were labeled, scale ranged from “very little” to “very high”), *valence* (from “very negative” to “very positive”), and *arousal* (from “not at all” to “extremely”). Food items were additionally rated on *palatability* (from “not at all” to “extremely”) and *desire to eat* (from “not at all” to “extremely”). General instructions read “how palatable is this food for you in general?” and “how much would you like to eat this food right now if it was in front of you.” Anchors on each visual analog scale for each image read “Palatability” (in German “Schmackhaftigkeit”): “not at all” to “extremely”; and “Desire to eat” (German “Verlangen”): “not at all” to “extremely.” Complexity (German “Komplexität”): “very low” to “very high”; was explained as being characterized by “many components, details and subobjects” as well as by “many edges and borders.” The VAS was displayed as a solid bar along which a cursor was to be moved; the rating was logged upon mouse click. The scale represented, invisible to the participants, 100 points (from 1 to 100).

### Data analyses

To describe and explore the *food-pics* normative database and to highlight some variables that might guide users during image selection and study design we performed the following analyses:

1. *Image type*: Descriptive data are given on stimulus valence and arousal across different stimulus classes (including non-food images) in the database. For foods (and most remaining analyses), palatability and desire to eat ratings are of prime importance and are reported as a function of *caloric content* (high- vs. low-calorie foods)*, sweetness* (sweet vs. savory foods) and *degree of processing* (whole vs. processed foods).
2. *Individual differences* and *demographics:* Effects of gender, age, and BMI, as well as diet (omnivore vs. vegetarian), and culture (German speaking vs. North American) were explored with regard to palatability and desire to eat ratings.
3. *State variables:* Hunger ratings were correlated with palatability and desire to eat ratings. Likewise, dieters (“current weight reduction diet”) were compared with non-dieters on palatability and desire to eat ratings.
4. *Image characteristics, ratings, and macronutrients*: Correlational analyses explored relationships between subjective ratings, image characteristics, and nutrients.

Generally, due to the high statistical power in the present sample, we only report effects with at least medium (η^2^ > 0.06, Cohen's *d* > 0.3) effect sizes unless otherwise noted. Within each subgroup of comparisons we used paired sample Student *t-test* to compare subgroups of images or display 95% confidence intervals.

## Results

### Image type

**Figure 2:**
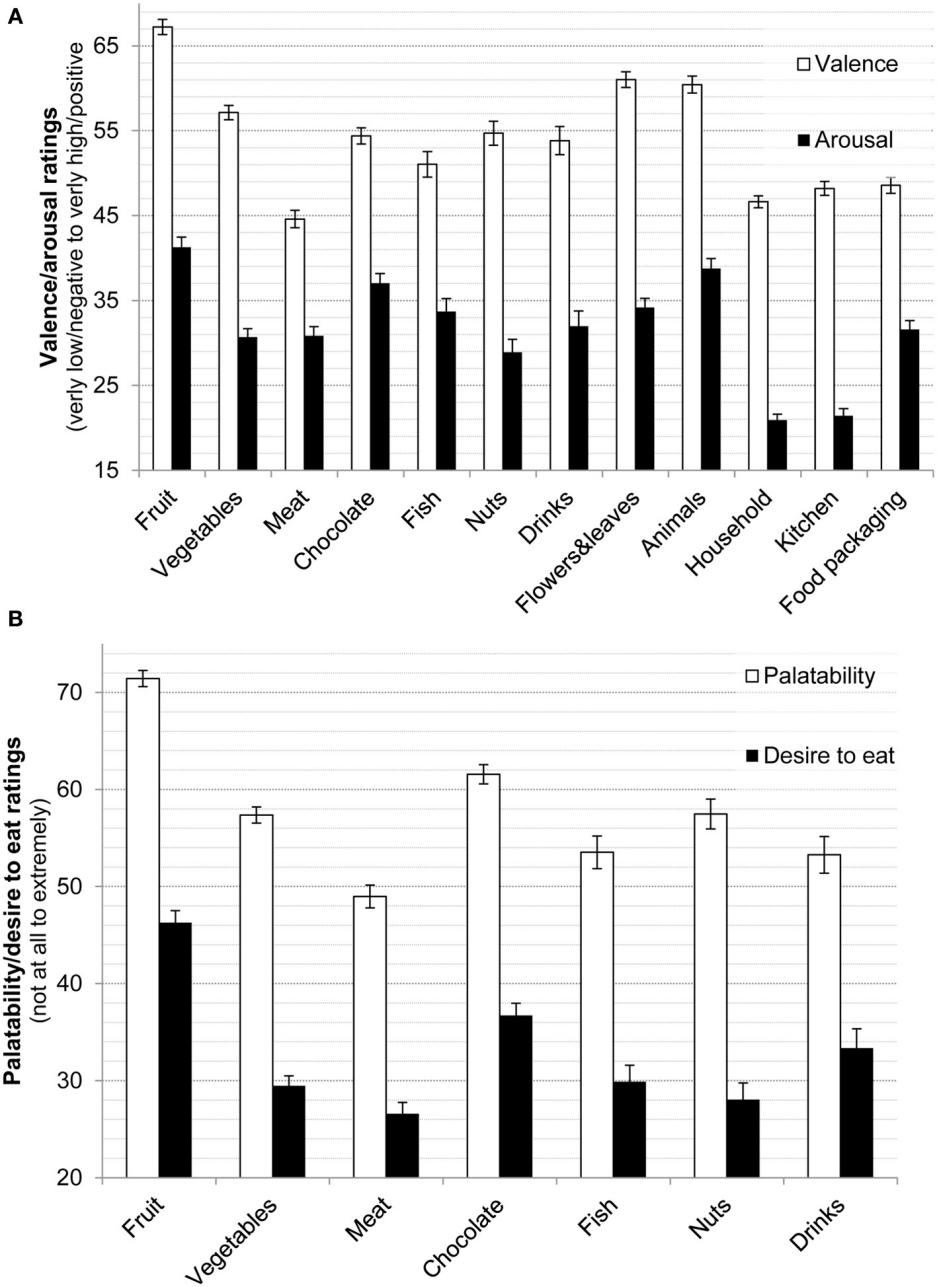
**(A)** Means and 95% confidence intervals for valence (“very negative” to “very positive”) and arousal (“very little” to “very high”) across all image categories. **(B)** Means and 95% confidence intervals for palatability and desire to eat (both “not at all” to “extremely”) across food types.

To provide an example characterization food and non-food objects were classified into several specific categories. Food objects were categorized, based on the dominant food in the image, into fruits (13.3% of all food images), vegetables (20.7%), chocolates (11.4%), meat (11.1%), fish (2.28%), nuts (1.76%), beverages (1.58%) and 38% other foods without clear dominance of one food type. Non-food images were categorized into flowers & leaves (13.4%), animals (10.1%), tools (7.32%), household items (non-kitchen, 28.3%), kitchen utensils (14.6%), office supply (6.37%), food packaging (10.5%) and other items (1%). Figure 2 displays valence, arousal, palatability, and desire to eat ratings for these categories along with 95% confidence intervals. Objects, flowers & leaves and animals were rated more positively on valence compared to tools, household and kitchen utensils as revealed by non-overlapping confidence intervals. Flowers & leaves and animals were also rated more positive on valence than most of the foods, except for fruit. Within foods, fruits were most popular, both in terms of valence and palatability and in terms of desire to eat. Interestingly, meat was rated lowest on palatability and desire to eat (closely followed by nuts for desire to eat).

In addition, as previous research has contrasted foods according to *caloric density, degree of processing, and gustatory qualities*, we classified our food pictures into high vs. low caloric density (median split regarding caloric density = kcal/100 g) as well as into processed (32.0% of all foods) vs. whole (66.7% of all foods, 1.3% not classifiable) and sweet (42.8%) vs. savory foods (38.8%, 18.4% not classifiable*;* see Table 2 for means and standard deviations of all ratings of the different food types) and determined palatability and desire to eat ratings for each category. *High vs. low calorie-dense* foods received lower ratings in terms of palatability, *t*_(1942)_ = 13.0, *p* < 0.001, *d* = 0.46, and desire to eat, *t*_(1942)_ = 9.3, *p* < 0.001, *d* = 0.42. *Sweet* vs. *savory* foods received higher ratings in terms of palatability, *t*_(1960)_ = 20.3, *p* < 0.001, *d* = 0.46, and desire to eat, *t*_(1960)_ = 18.8, *p* < 0.001, *d* = 0.42. *Whole* vs. *processed* foods received higher ratings in terms of palatability, [*t*_(1858)_ = 15.1, *p* < 0.001, *d* = 0.35] and desire to eat, [*t*_(1858)_ = 9.86, *p* < 0.001, *d* = 0.23]. In brief, valence and arousal ratings largely mirrored these differences and familiarity and recognizability was consistently high (>93.2% of all foods were rated as recognizable and 94.6% of all foods were rated as familiar).

**Table 2:** **Subjective ratings as a function if different food types (mean, standard deviations)**.

	**High calorie**	**Low calorie**	**Processed**	**Whole**	**Sweet**	**Savory**
Palatability	56.8 (16.8)	60.9 (15.4)	57 (16.1)	62.8 (17)	62.2 (16.6)	55.5 (16.4)
Desire to eat	32 (21.5)	35.1 (20.7)	32.4 (21)	36.1 (21.7)	37.5 (22.5)	30.4 (21)
Valence	52 (16.7)	58.1 (16.2)	52.3 (16.1)	61.2 (18.3)	56.9 (16.8)	52.2 (15.7)
Arousal	33.4 (20.9)	34.7 (21.4)	33.9 (20.6)	34.6 (22.6)	37 (22.3)	32.2 (20.4)
Recognizability (%)	94.6 (8.72)	96.1 (9.30)	93.2 (9.25)	96.4 (7.3)	94.4 (9.27)	93.4 (10.4)
Familiarity (%)	94.6 (8.73)	96.2 (9.35)	95.1 (8.42)	97.6 (6.9)	95.2 (9.58)	96.1 (8.66)

Recognizability and Familiarity were dichotomous yes/no decisions.

### Influence of demographics and individual difference variables: culture, gender and vegetarianism, BMI and age

In brief, effects of culture (North America vs. German speaking) on all food ratings (all foods, high calorie vs. low calorie/processed vs. non-processed foods, meat vs. non-meat) were significant but of small effect size (η^2^_*p*_ < 0.06) when considering age and gender differences between the samples as covariates.

*Women* gave lower desire to eat ratings for all foods compared to men [*M* = 32.2, *SD* = 19.6 vs. *M* = 40.5, *SD* = 20.5, *t*_(1963)_ = 7.70, *p* < 0.001, *d* = 0.42] whereas no gender differences were found for palatability [*M* = 58.8, *SD* = 14.5 vs. *M* = 59.1, *SD* = 15.6, *t*_(1963)_ < 1.00].

V*egetarians* rated meat containing images lower than omnivores on palatability [*M* = 19.6, *SD* = 21.2, vs. *M* = 56.1, *SD* = 21.2, *t*_(1879)_ = 29.6, *p* < 0.001, *d* = 1.72] and desire to eat [*M* = 7.46, *SD* = 13.2, vs. *M* = 31.3, *SD* = 26.3, *t*_(1879)_ = 16.8, *p* < 0.001, *d* = 1.21].

*BMI* was not associated with palatability [*r*_(1916)_ = 0.029, *n.s.*] and positively but weakly correlated with desire to eat [*r*_(1961)_ = 0.117, *p* < 0.001, for high-calorie foods, *r*_(1961)_ = 0.146, *p* < 0.001, for low calorie foods *r*_(1961)_ = 0.059, *p* < 0.001]. Correlations of *age* with palatability and desire to eat were very weak (*r*s < 0.1).

### Influence of state variables: hunger and current dieting

Interestingly, being currently on a weight reduction diet (13.6% answered this question with yes) did influence ratings only to a minor degree. Dieters did not differ from non-dieters on palatability ratings [*M* = 59.0, *SD* = 14.9, vs. *M* = 58.8, *SD* = 14.8, *t*_(1963)_ < 1.00] and gave slightly elevated desire to eat ratings [*M* = 37.1, *SD* = 19.9, vs. *M* = 33.5, *SD* = 20.1, *t*_(1963)_ = 2.71, *p* = 0.007, *d* = 0.21]. Hunger (averaged across pre- and post-questionnaire ratings) was weakly positively correlated with palatability, *r*_(1965)_ = 0.120, *p* < 0.001 (*r* = 0.04 and *r* = 0.141 for low- and high-calorie food images, respectively), but showed a medium sized positive correlation with desire to eat, *r*_(1965)_ = 0.528, *p* < 0.001 (*r* = 0.473 and *r* = 0.524 for low- and high-calorie food images, respectively).

### Image characteristics, ratings and macronutrients

The main purpose of including image characteristics was to allow for matching of different stimulus sets in studies using neurocognitive measures (e.g., set of high and low calories, i.e., Toepel et al., 2009). Since we had no neurocognitive measures in this database, we explored how image characteristics were related to (a) the subjective ratings and (b) macronutrients of the displayed foods. Such data could serve to raise awareness of the importance to control for such characteristics by an appropriate selection of images in future research. To do so, we computed Pearson correlations (images on rows) between picture characteristics and subjective ratings (averaged across all participants) as well as with macronutrients.

The only correlation of close to medium size indicated that image with stronger contribution of the red color channel were rated as more arousing, *r*_(883)_ = 0.279, *p* < 0.001. In addition, image complexity (edge detection), as well as normalized image complexity (complexity relative to image size) correlated with subjectively rated complexity [*r*_(883)_ = 0.349, *p* < 0.001 and *r*_(883)_ = 0.248, *p* < 0.001]. A higher contribution of the green color channel went along with lower concentrations of protein, fat and carbohydrates as well as with lower number of calories (*r* = −0.251, *r* = −0.209, *r* = −0.257, and *r* = −0.313, respectively)

## Discussion

The present study presents *food-pics*, a database of images of foods for experimental research on food perception and eating behavior. Previous studies are limited considerably in stimulus selection and/or characterization of stimulus material and food contents hampering the comparability of findings across laboratories. *Food-pics* comprises a large variety of foods and non-foods along with detailed data on image characteristics, food contents, and normative ratings. We presented example analyses of food types, individual differences, state effects, and image characteristics to explore key variables relevant for experimental design of food viewing studies.

Regarding *food types*, our results confirm that calorie content is a relevant determinant of subjective responses, in line with a several studies showing distinct neural responses for high- vs. low-calorie images (e.g., Killgore et al., 2003; Cornier et al., 2007; Toepel et al., 2009; Frank et al., 2010). Interestingly, our normative data suggest slightly lower palatability and desire to eat ratings for high-calorie images (small to medium effect size), possibly reflecting the rising awareness of the unhealthy nature of these foods in the populations studied here or self-presentation biases. Other self-report studies show the opposite (Richter et al., under review), as do implicit measures (Houben et al., 2012). It is possible that food restrictions prior testing played a role here because food deprivation renders particularly high-calorie foods more attractive (Goldstone et al., 2009). Our data indicate that participants were not very hungry [*M* = 28.5, *SD* = 25.4, on a 1 (not hungry) to 100 (very hungry) scale] but hunger correlated slightly stronger with palatability/desire to eat ratings of high- compared to low-calorie images. Sweet compared to savory foods were rated more palatable and with stronger desire to eat, as were whole vs. processed foods. One has to keep in mind that we used all images of the respective type of the database so it might well be that certain subcategories with a high number of images contributed more than others (e.g., 76 images displayed fruit in the whole and sweet categories). Together these results suggest that image selection will substantially influence (rating) results, depending on the proportion of high-calorie, sweet and whole foods in a specific category. Processed foods are often higher in caloric density, however, researchers could still match the total amount of calories displayed in the images between whole and processed foods by selecting pictures with larger amounts of whole foods (e.g., wild berry mix, 53,75 kcal, image #214) and pictures with smaller amounts of processed foods (e.g., 4 pretzels, 44 kcal, #494). Although recognizability and familiarity of the objects were relatively high, it should be noted that participants performed a yes/no task and did not name the objects.

*Individual differences* such as restraint, external or emotional eating, eating disorders, or obesity are central independent variables in the study of eating behaviors. However, sampling error can induce group differences on other individual difference variables unless carefully stratified. Age and BMI differences are tolerable to some degree because they showed only minor influence on ratings in our analyses (*r*s < 0.117). Gender and vegetarianism are more relevant for sampling/matching because lower ratings for palatability and desire to eat were found for women in general and for vegetarians specifically for meat-containing foods. These results reflect in part also inconsistencies in the literature with regard to gender: women are sometimes reported to experience cravings more frequently (Cepeda-Benito et al., 2003) but also restrain and worry about their eating more than men (Dinkel et al., 2005). The present data suggest that in a large unselected sample and across a wide range of foods, women give lower palatability/desire to eat ratings. Thus, normative ratings provided along with the images are reported separately for vegetarians and omnivores and for males and females to facilitate selection of suitable images.

*State variables* like hunger are obviously important in the food context. Hunger influenced desire to eat to a higher degree than palatability, which is in line with findings that specific state cravings correlate with food deprivation (Cepeda-Benito et al., 2003; Meule et al., 2012) and interesting in the context of the discussion whether “wanting” (~desire to eat) and “liking” (~palatability) are dissociable in humans (Finlayson et al., 2007; Havermans et al., 2009; Finlayson and Dalton, 2011; Havermans, 2011). Hunger might further interact with caloric density as discussed above. Interestingly, current dieting did not influence results much: only a small increase in desire to eat was found for dieters as compared to non-dieters. The literature on dieting effects is mixed: some studies have found dieting to decrease food cravings (reviewed in Martin et al., 2011) other studies found the opposite (Massey and Hill, 2012). On the other hand, weight reduction has been reported to lead to long term weight gain (so called “yoyo effects”) although the mechanisms are not clear (Ochner et al., 2013). These findings underscore the necessity to assess the short and long-term dieting status, the diet success (i.e., weight reductions) as well as the current hunger levels of the participants.

Just as the appearance of foods influences their acceptability for consumption (Wadhera and Capaldi-Phillips, 2014), *image characteristics* have been shown to affect neurocognitive measures (Vuilleumier et al., 2003; Bradley et al., 2007; Delplanque et al., 2007; Thierry et al., 2007; Rossion and Jacques, 2008) which is why we calculated the key measures that have been established in the field of visual perception (Knebel et al., 2008; Willenbockel et al., 2010; Wiens et al., 2011; Kovalenko et al., 2012; Foroni et al., 2013). Lacking neurocognitive measures in the present study, we explored their relationship with normative ratings and macronutrients. Red color went along with higher arousal ratings whereas green color was indicative of lower calories and lower concentrations of protein, fat, and carbohydrates. Colors should therefore be considered in the study design. Expectedly, our objective index of complexity (reflecting the number of object-components displayed in the image) correlated positively with rated complexity. However, the low to medium sized correlation indicates that subjective and objective measures of complexity are partially independent constructs and studies need to make their pick of which index to use depending on study aims. Future studies might further measure image aesthetics which was not measured here but might be related to expected palatability. Further research should also employ neurocognitive measures to determine which objective and subjective image characteristics influence neural responses. In the lack of such evidence, researchers could use *food-pics* metadata to match image sets on factors unrelated to their independent variable, particularly when comparing different food types against each other. For example, if the influence of caloric density is to be examined, high- and low-caloric density image sets could be matched for total amount of calories in the image, sweet/savory and processed/whole food proportion, and green color contribution to increase the specificity of the comparisons. If matching is not possible or not desired, researchers should still describe their images in more detail using the metadata provided with *food-pics* or list the image numbers in a footnote or supplementary material.

In conclusion, we hope that *food-pics* will facilitate experimental research on food perception, eating behavior and appetitive responses. Databases such as *food-pics* will increase the comparability of study results and therefore facilitate research communication as it is the case in object recognition, face processing or emotional picture viewing. *Food-pics* as well as normative rating data can be downloaded free of charge from the first author's website at www.food-pics.sbg.ac.at upon completion of appropriate license agreements.

### Conflict of interest statement

The authors declare that the research was conducted in the absence of any commercial or financial relationships that could be construed as a potential conflict of interest.

## Footnotes

^1^Image numbers were 4, 9, 10, 15, 26, 41, 46, 64, 85, 95, 101, 110, 116, 134, 148, 152, 153, 159, 185, 189, 192, 193, 194, 198, 199, 205, 206, 211, 244, 248, 249, 262, 264, 265, 282, 298, 308, 309.
